# Maintaining Balance when Looking at a Virtual Reality Three-Dimensional Display of a Field of Moving Dots or at a Virtual Reality Scene

**DOI:** 10.3389/fneur.2015.00164

**Published:** 2015-07-27

**Authors:** Elodie Chiarovano, Catherine de Waele, Hamish G. MacDougall, Stephen J. Rogers, Ann M. Burgess, Ian S. Curthoys

**Affiliations:** ^1^CNRS UMR 8257, Cognition and Action Group, Centre Universitaire des Saints-Pères, Université Paris Descartes, Paris, France; ^2^Vestibular Research Laboratory, School of Psychology, University of Sydney, Sydney, NSW, Australia

**Keywords:** vestibular, proprioception, balance, posture, virtual reality, optokinetic

## Abstract

**Experimental objective:**

To provide a safe, simple, relatively inexpensive, fast, accurate way of quantifying balance performance either in isolation, or in the face of challenges provided by 3D high definition moving visual stimuli as well as by the proprioceptive challenge from standing on a foam pad. This method uses the new technology of the Wii balance board to measure postural stability during powerful, realistic visual challenges from immersive virtual reality.

**Limitations of current techniques:**

Present computerized methods for measuring postural stability are large, complex, slow, and expensive, and do not allow for testing the response to realistic visual challenges.

**Protocol:**

Subjects stand on a 6 cm thick, firm, foam pad on a Wii balance board. They wear a fast, high resolution, low persistence, virtual reality head set (Oculus Rift DK2). This allows displays of varying speed, direction, depth, and complexity to be delivered. The subject experiences a visual illusion of real objects fixed relative to the world, and any of these displays can be perturbed in an unpredictable fashion. A special app (BalanceRite) used the same procedures for analyzing postural analysis as used by the Equitest.

**Power of the technique:**

Four simple “proof of concept” experiments demonstrate that this technique matches the gold standard Equitest in terms of the measurement of postural stability but goes beyond the Equitest by measuring stability in the face of visual challenges, which are so powerful that even healthy subjects fall. The response to these challenges presents an opportunity for predicting falls and for rehabilitation of seniors and patients with poor postural stability.

**Significance for the field:**

This new method provides a simpler, quicker, cheaper method of measurement than the Equitest. It may provide a new mode of training to prevent falls, by maintaining postural stability in the face of visual and proprioceptive challenges similar to those encountered in life.

## Introduction

Falls are a major problem for seniors, with significant morbidity and mortality. Major physical injuries are relatively uncommon but are often associated with ongoing disability. Psychological sequelae such as fear of falling occur frequently and may lead to increased dependency. There are physical, psychosocial, and financial costs to the injured person. The cause of falls is usually multifactorial involving combination of age-related sensory, neural and muscular decline, chronic disease, medication, and environmental hazards. A multidisciplinary approach is often indicated for assessment and treatment of people who repeatedly fall.

The integration of the somatosensory, vestibular, and visual systems is involved in maintenance of balance. In patients, balance has been studied using classical posturographic platforms or Equitest (Neurocom, Clackamas, OR, USA), which included a Sensory Organization Test (SOT) and a motor test. The SOT allows quantifying the weight of different vestibular, visual, and proprioceptive inputs in maintaining balance (1). However, the high cost of the Equitest machine and the fact that it only allows the study of balance in one plane, the sagittal plane, has limited its attractiveness. Therefore, we aimed to develop a new low-cost system using new technologies to quantify balance objectively using the Wii Balance Board (WBB, Nintendo, Kyoto, Japan) alone or the WBB plus foam rubber pad (WBB + f). This simple system allows for the study of balance in all planes. WBB has been extensively used in the last 10 years to quantify balance (2), but only a few published studies have measured balance with the subjects standing on a foam pad located on the WBB (3, 4). WBB + f provides a challenge such that proprioceptive input is continuously modified as the subject seeks to maintain balance (5). In this condition, we have developed new technology and new programs to present high-resolution, wide-field, three-dimensional dynamic visual stimuli.

The display in the virtual reality (VR) goggles is continuously modified by the head position, as detected by inertial sensors in the goggles. Images move across the display screen in the opposite direction to head movements, so the subject experiences a visual illusion of real objects fixed relative to the world. The subjective experience is the perception that the subject is immersed inside the field. This is in contrast to a display which is not adaptively changed according to head movement: with the latter the result is that as the head moves the display remains in the same direction with respect to the head, and the perception of immersion within the field is absent. Instead of a stationary scene, we have used a 3D field of moving dots and a separate VR scene to quantify the effect such realistic visual distractors have on the maintenance of balance during this continuous challenge to proprioception from standing on the foam pad. While these full-field visual stimuli are “optokinetic” in the sense that they are moving visual stimuli, we prefer the term “distractor” to describe them since they are extremely realistic and are subjectively much more powerful than the usual 2D moving patterns used in studies of visual-vestibular interaction. The term “distractor” also appears to be especially appropriate in the case of the VR display, where the scene was unpredictably rotated and so was “distracting” in the sense used in studies of attention (6).

Poor quality VR has been available for some years, but it was slow and had a long persistence. Until recently adapting a low-persistence, high-resolution image at high speed according to head movements has not been available. The newly released Oculus Rift DK2 (Oculus, Menlo Park, CA, USA) satisfies these requirements.

As a “proof of concept,” we have quantified the balance performance of healthy subjects using this new system and compared their results on WBB + f to their results on Equitest platform testing. In this way, we sought to validate our new measures against the gold standard Equitest platform and then to extend the application of WBB + f into new domains.

Our aims were twofold:

(1) to understand the weighting of proprioceptive and vestibular inputs in maintaining balance by using the WBB and WBB + f in eyes closed condition – thus eliminating visual input;
(2) to provide a new method using VR to answer the question: is maintaining balance an automatic process, or is maintaining balance a challenging task requiring attentional demands? We suggested that some people may lose their ability to maintain automatic balance, when they have eyes closed or are confronted by visual distractors. Using this new method of studying the effect of distractors on balance may be a way of better understanding the attentional demands required to stand upright.

Moreover, we suggest that the response of subjects with postural instability to these powerful visual stimuli, which are visual distractors in an attentional sense (6) may be a predictor of falls in the medium term, because of results on a parallel “dual task” attentional paradigm that has been used in studies of gait to predict falls. It was shown that aging people stopped walking when talking because of an overload of attentional demands (7, 8).

## Materials and Methods

All test subjects were healthy, active, community dwelling individuals with no record of vestibular or central pathologies. They gave informed consent. The procedures followed were in accordance with the ethical standards of the Helsinki Declaration and were approved by the University of Sydney Human Ethics Committee – Protocol number 2013/288.

One hundred sixty one healthy subjects (mean age: 56.7 ± 18 years, range 21–92 years, 76 females and 85 males) were recorded on the WBB and WBB + f in eyes open and eyes closed conditions.

Twenty seven of these subjects were studied to compare the data obtained with the WBB and with the Equitest machine as the gold standard platform. We used two visual conditions (eyes open and eyes closed) and two surface conditions (standing upright on the platform or on the platform plus foam).

In addition, nine subjects were studied with visual distractors. They were asked to maintain balance during 25 s in different conditions:

1. on the WBB without foam: (a) eyes open, (b) eyes closed, (c) looking at a VR three-dimensional visual scene, and (d) looking at a VR three-dimensional field of dots moving in one particular trajectory (optokinetic stimuli);
2. on the WBB with foam: (a) eyes open, (b) eyes closed, (c) looking at a VR three-dimensional visual scene, and (d) looking at a VR three-dimensional field of dots moving in one particular trajectory (optokinetic stimuli).

The voltage output from the WBB with a mean sample rate of 97.4 ± 1.8 Hz was recorded by an iPod touch (with a customized app). The materials consisted of an Oculus Rift DK2 head mounted display system, a safety nacelle, and the foam rubber pad (Airex Balance Pad (Airex AG, Sins, Switzerland) – 41 cm × 50 cm × 6 cm thick). In this study, even healthy subjects fell, but safety was ensured by the closely adjacent rails of the nacelle, at about the hand height of the standing subject, and because one experimenter stood behind the subject. We deliberately did not use a harness since the somatosensory cues from that harness may provide a reference, which can interfere with the measured balance performance. The safety harness was not used in testing with the Equitest platform. If any part of the body touched the nacelle or if the subject required support by the operator, the trial was recorded as a fall.

The visual stimuli were generated by a custom program in LabVIEW (version 2012; National Instruments, Austin, TX, USA) and delivered through the Oculus Rift DK2, which is a significant improvement over the DK1 with higher screen resolution and with a faster response time (shorter persistence). The display was 1920 pixels wide × 1080 pixels high (960 × 1080 per eye) with a 100° field of view.

The VR scene was a moving visual scene of a house and garden, sky and sea (modified from the “Oculus Tuscany Demo” developed by FenixFire and Oculus VR – Irvine, CA, USA). With our custom program this scene could be rotated around pitch, roll or yaw axes, and during testing the scene was unpredictably rotated by a sum-of-sines pseudorandom waveform, which drove each axis. The *X* axis rotation was the sum of three sine waves with frequencies of 0.5, 0.2, and 0.1 Hz and zero phase. The *Y* axis rotation was the sum of three sine waves with frequencies of 0.4, 0.1, and 0.1 Hz and phase angles of 0, 25, and 0°. The *Z* axis rotation was the sum of three sine waves with frequencies of 0.5, 0.2, and 0.2 Hz and phase angles of 70°, 45°, and 90°. Peak amplitude of the rotation was fixed by the experimenter at arbitrary values. In this study, peak amplitudes used were arbitrary values of 0.1, 0.2, 0.5, and 1.0, where peak amplitude of the arbitrary value of 1.0 produced a maximal rotation of 30°.

**Figure 1:**
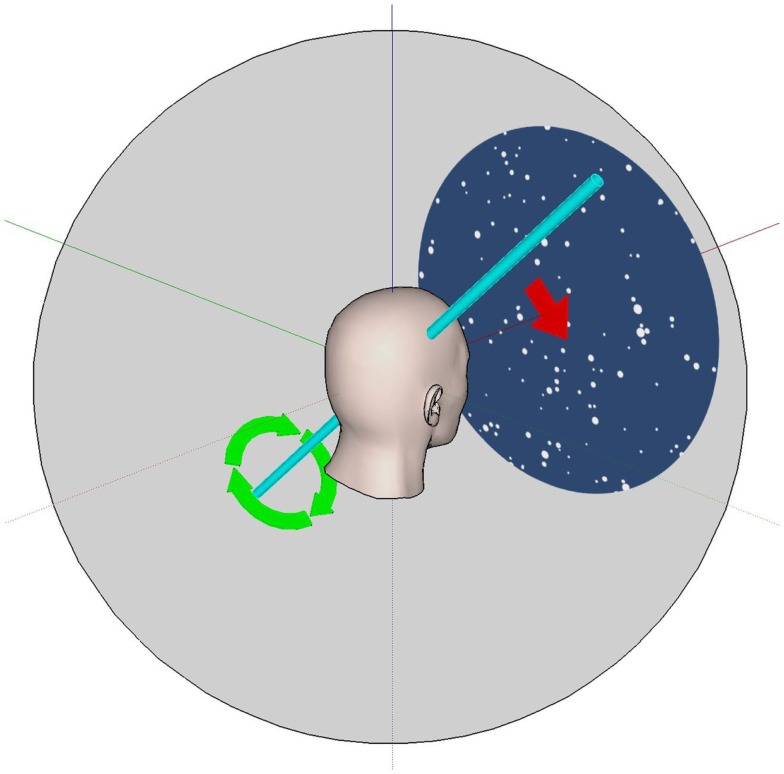
**The VR optokinetic stimulus in an oblique plane**. The white dots visible to the subject appear to move as if they were attached to a sphere (gray) that rotates about an oblique axis. This axis (shown in light blue) is halfway between the *Y* (pitch) and *Z* (yaw) head axes; all axes here are attached to (i.e., they move with) the head. For example, positive rotation (in the direction of the green arrows) about the light blue axis leads to dots which appear to move diagonally down and to the right (red arrow). Negative rotation about this axis leads to dots that move up and to the left.

The optokinetic (OKN) stimulus was a display of 1000 spheres of 10 cm radius, randomly distributed in 3D around a head-centered globe and programed to appear to lie in depth between 10 and 50 m from the view point. The screen background was blue (RGB levels on a scale from 0 to 255 were R:49, G:77, B:121) and the spheres were white (R:255, G:255, B:255). Every 200 ms a random sphere was deleted and a new one created in a random location so that the pattern was always changing and so was unrecognizable. The subjective experience was like being inside a 3D planetarium. In the present study, the visual stimulus was rotated around the subject at a rate of 20°/s. The entire three-dimensional field (globe with all spheres) was rotated at a velocity of 20°/s and around different axes (Figure 1): so that the dots appeared to move in the horizontal plane (left and right direction), in the vertical plane (up and down directions), in the frontal plane (torsional clockwise and counter-clockwise directions), and in an oblique plane (i.e., diagonal or simultaneous combinations of horizontal and vertical to be approximately in the planes of two of the vertical semicircular canals: rotation was around the axis *z* = −*y* (Hixson) relative to the subject’s head position). The values chosen for these parameters were based on preliminary observations and may not be optimal – further testing is required to optimize the stimuli but here we simply wanted to test whether this concept was workable. A movie depicting an example visual stimulus is provided as Supplementary Material.

The order of testing was the same for all subjects. The testing session started on the WBB with eyes open, then eyes closed, and then the two visual stimuli. The order of the visual stimuli (VR and moving dots) was randomized. The same visual conditions (in the same order) were repeated on the WBB + f.

**Figure 2:**
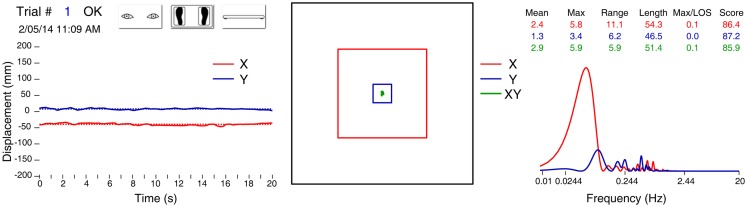
**Data report for one trial of a healthy 60-year-old male, on foam, eyes open, no visual distractors**. Left panel: position of the center of gravity in *X* (red trace) and *Y* (blue trace) axis. Middle panel: statokinesigram (green trace), limit of normality (small blue square), and limit of stability (larger red square). On the right: table of several variables and graph of the Fourier analysis.

Performance of the subject was assessed by measuring path length (in mm) *X* (interaural), *Y* (nasooccipital), and *XY* (diagonal); and velocity (in *X*, *Y*, and *XY* in cm/s) of the center of gravity of the body estimated from values obtained with the center of pressure of the feet (Figure 2). COP measurements were standardized by calculation of a score (in %). For each COP measurement, a value between 0 and 1 was calculated according to the formula:

$$v_{\text{i}}=1-\left(\frac{d_{\text{i}}}{D}\right)$$

where *d*_i_ is the distance from the COP measurement to the center (upright) position, and *D* is the distance from the subject’s limit of stability to the center position. The value of *v*_i_ for a COP measurement placed on the center was 1; for a COP measurement placed on the limit of stability *v*_i_ was 0; and for a COP measurement placed at 3/4 of the distance to the limit of stability *v*_i_ was 0.25. The individual values of *v*_i_ over the whole time series were averaged to give the score. These methods have been explained in detail in an earlier paper (9), and here they were implemented in an app running on the iPod Touch. Two criteria quantified a fall: presence or absence, and the elapsed time before the fall (in seconds).

The statistical analysis was performed with SAS 9.4 software. Bland and Altman plots (10) and Pearson’s correlation coefficient were used to assess the agreement between WBB and Equitest platform.

## Results

### Experiment 1: Balance on the WBB and WBB + f with eyes open and eyes closed

The average values for 161 subjects for the measured parameters in the four conditions are shown in Table 1. No difference was found between *X* length and *Y* length and between *X* velocity and *Y* velocity over the four conditions (Table 1), thus we focus on *XY* length and *XY* velocity in cm/s only.

**Table 1:** **Means ± SDs of *XY*, *X*, and *Y* velocity (cm/s) and length (cm) for the four conditions (WBB or WBB + f, eyes open or closed)**.

	WBB		WBB + f	
	Eyes open	Eyes closed	Eyes open	Eyes closed
*XY* velocity	0.56 ± 0.18	0.76 ± 0.33	1.28 ± 0.54	3.45 ± 1.20
*X* velocity	0.38 ± 0.15	0.55 ± 0.23	0.84 ± 0.40	2.30 ± 0.83
*Y* velocity	0.32 ± 0.13	0.40 ± 0.23	0.79 ± 0.35	2.08 ± 0.88
*XY* length	10.11 ± 3.32	13.70 ± 5.88	23.03 ± 9.73	59.65 ± 18.91
*X* length	6.93 ± 2.54	9.99 ± 3.97	15.12 ± 7.16	39.84 ± 13.35
*Y* length	5.85 ± 2.27	7.24 ± 4.05	14.16 ± 6.16	35.85 ± 13.12

*Falls occurred in only in those healthy subjects older than 65 years (*n* = 11, i.e., 7% of the 161 subjects), and these subjects fell only in the condition WBB + f with eyes closed*.

**Figure 3:**
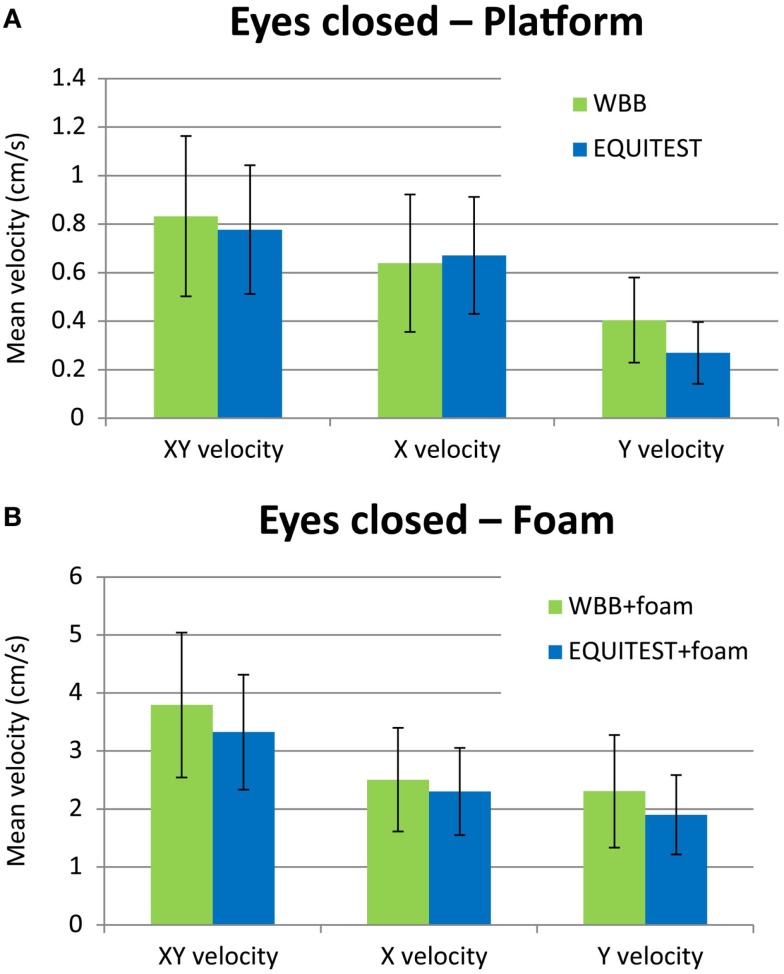
**Histogram of the mean *XY* velocity in condition eyes closed on the WBB (green bars) and on the Equitest platform (blue bars) without foam (A) and with foam (B)**. The error bars show the SD. Note that **(B)** has a much larger vertical scale than **(A)**, because of the larger deviations in posture when standing on foam.

**Figure 4:**
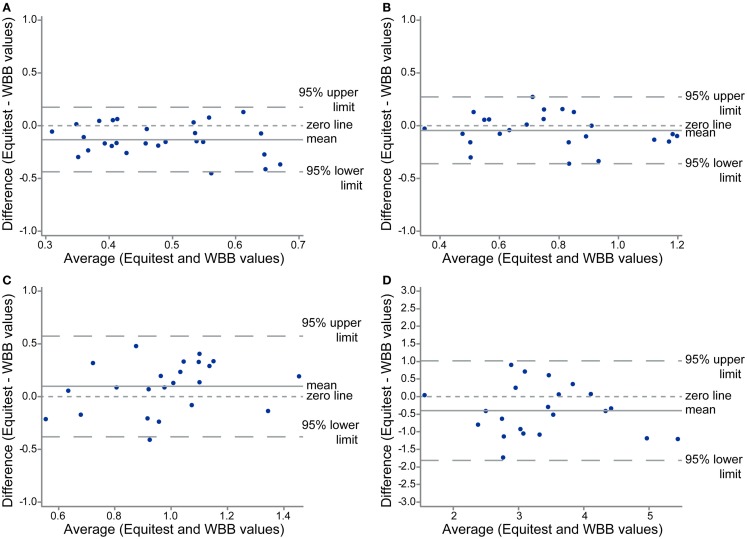
**Bland and Altman plots of the *XY* velocity for the four conditions: eyes open on platform (A), eyes closed on platform (B), eyes open on the foam (C), and eyes closed on the foam (D), showing agreement between the two set of measures: with the Equitest platform and the WBB**.

In 27 subjects, we compared the data obtained with the WBB and with the Equitest platform in conditions eyes open and eyes closed, without and with foam (Figure 3). The Bland and Altman plots of the *XY* velocity are shown in Figure 4, and the mean values of the *XY* velocity for the WBB and the Equitest platform are given in Table 2. These plots show a strong agreement between Equitest platform and WBB measurements. Moreover, the two sets of measures were significantly (and linearly) correlated for the four conditions (Pearson’s correlation coefficient, *P* < 0.05; details in Table 2). Results of the analysis are here shown for the *XY* velocities, but the correlations between Equitest platform and WBB measures were significant for both the *X* and the *Y* velocities as well.

**Table 2:** **Means ± SDs of *XY* velocity values obtained on the Equitest platform and on the WBB; and mean ± SD of the difference (Equitest value – WBB value)**.

	Eyes open, platform	Eyes closed, platform	Eyes open, foam	Eyes closed, foam
Difference (cm/s)	−0.13 ± 0.15	−0.04 ± 0.15	0.09 ± 0.23	−0.39 ± 0.70
Equitest (cm/s)	0.41 ± 0.11	0.74 ± 0.23	1.02 ± 0.26	3.17 ± 0.90
WBB (cm/s)	0.54 ± 0.14	0.78 ± 0.26	0.92 ± 0.21	3.57 ± 0.99
Pearson’s *r*	0.47	0.80	0.53	0.72
*P*	0.01	<0.0001	0.009	0.0001

*Last two rows: Pearson’s correlation coefficient *r* for the agreement between Equitest platform and WBB, and the associated probability*.

### Experiment 2: Balance on the WBB and WBB + f with virtual reality visual distractor

In this “proof of concept” study, we measured the performance of nine healthy subjects (mean age 45.5 ± 19 years; range 18–76 years, five females and four males) as the amplitude of the visual distractor perturbation was increased, while the subject stood on the WBB or on the WBB + f.

**Figure 5:**
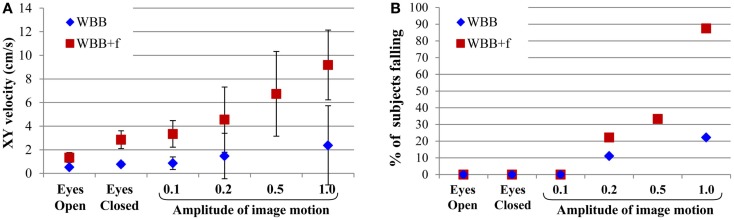
**(A)** Velocity in *XY* of body center of gravity displacement for the visual conditions delivered (eyes open and closed, and virtual reality with amplitudes of 0.1, 0.2, 0.5, and 1.0). The error bars show the SD. **(B)** Percentage of subjects falling depending on the intensity of moving scene with eyes open and closed, as well as during stimulation using virtual reality.

The velocity of the displacement of the center of gravity and the percentage of falls increased with the amplitude of the perturbation (Figure 5). This effect was greater on the WBB + f (red squares) than on the WBB (blue diamonds). The average time to fall on the WBB was between 6.7 and 13.4 s; and on the WBB + f was between 5.5 and 20.5 s.

The arbitrary amplitude of 0.1 is particularly interesting because it did not induce falls in healthy subjects, and because with the subject standing on WBB + f, the velocity of the displacement of the center of gravity was similar in the eyes-closed condition to the condition of VR with amplitude of 0.1. Our hypothesis is that in seniors and vestibular patients suffering dizziness this very modest stimulus will induce a fall.

### Experiment 3: Balance on the WBB and on WBB + f with moving dots optokinetic stimulation

**Figure 6:**
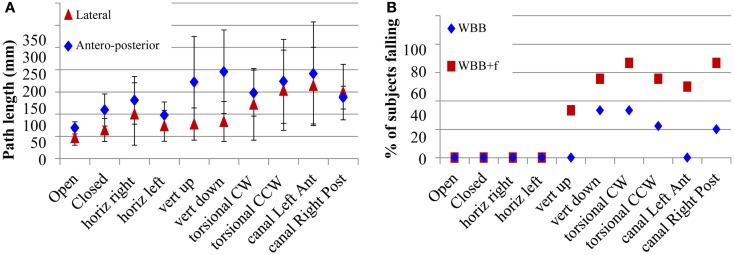
**(A)** Path length in *XY* of body center of gravity displacement for the different optokinetic stimulus conditions delivered (Horizontal, vertical up and down, torsional CW and CCW, oblique) on the WBB without foam. The error bars show the SD. **(B)** Percentage of subjects falling depending on the intensity of moving scene during stimulation using optokinetic stimuli.

For the same nine healthy subjects the VR 3D field of moving dots on the WBB induced a progressive displacement of the center of gravity of the body: forward displacement when dots were moving vertically downward; backward displacement when dots were moving vertically upward; rightward or leftward displacement when dots were moving in a torsional clockwise or counter-clockwise direction; and diagonal displacement (i.e., in the same direction as the direction of the stimulation) when dots were moving in the diagonal leftwards or rightwards directions (Figure 6). The only exception was the horizontal movement where there was no systematic effect. This is a very powerful optokinetic stimulus, which has allowed us to assess the sensitivity of subjects to postural challenges induced by moving optokinetic dots. On the WBB + f, the effect of the displacement direction was less visible because of body oscillations induced on the foam pad, which mask the direction of displacement of the center of gravity of the body. However, moving dots can induce falls at different times between 5.3 and 21.8 s on the WBB and between 3.2 and 25 s on the WBB + f after the start of the optokinetic stimulation.

### Experiment 4: Habituation and rehabilitation

**Figure 7:**
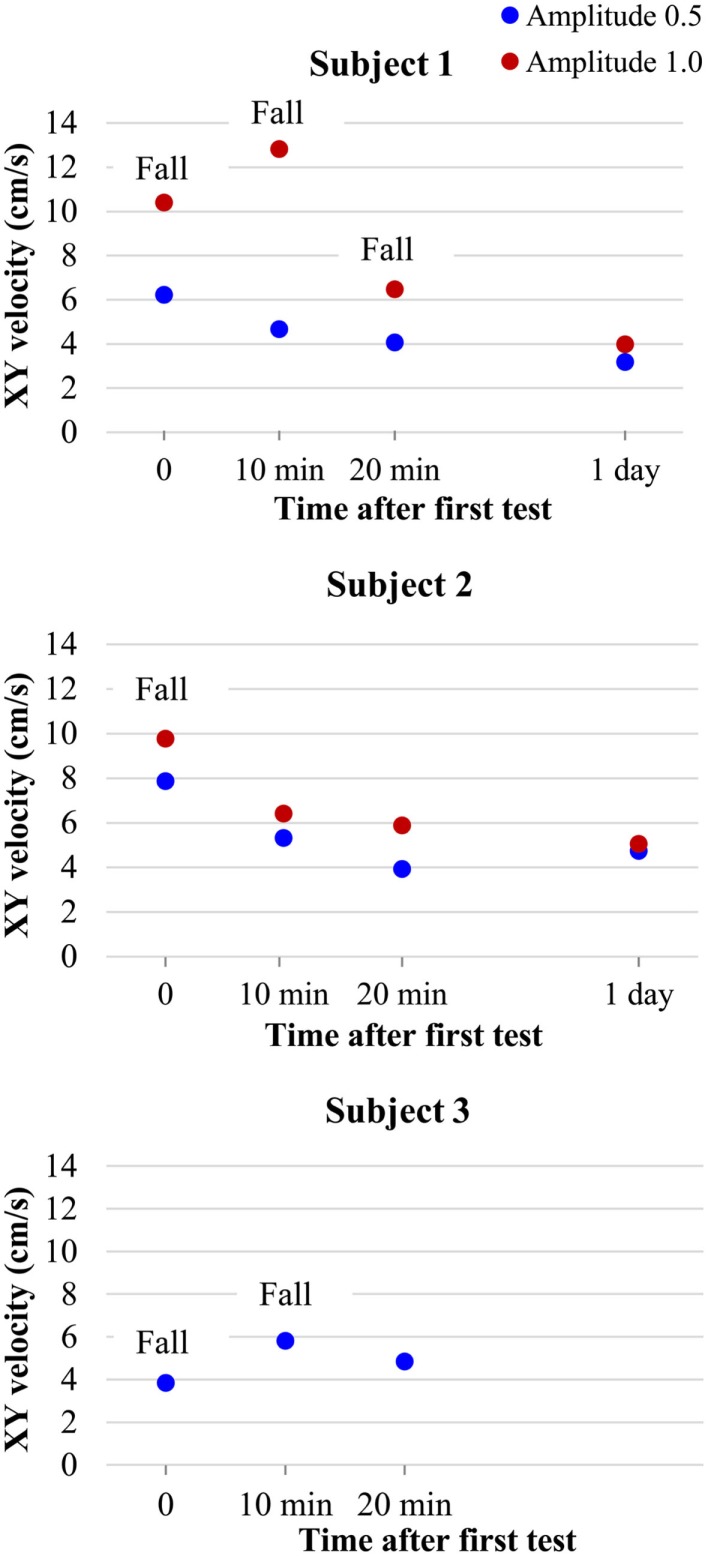
**Balance results of subjects tested on WBB + f with VR visual distraction at amplitudes of 0.5 (blue circles) and 1.0 (red circles) during continuous stimulation on WBB + f at several times (10, 20 min), during the habituation period and 1 day after**.

In addition, three subjects were tested for habituation to the VR stimulus. The VR stimulus at an amplitude of 0.5 was continuously presented during 1 h and the subject was tested every 10 min on the foam pad with the amplitude at 0.5 and 1.0. We observed that after 20 min of habituation, all subjects had improved, compared to the initial test – they showed decreased oscillation and/or no falls. This improved post-habituation performance could still be observed 1 day after habituation (Figure 7). These results have to be validated and reproduced in more subjects, but it appears this may be a valuable new method for rehabilitation to minimize the risk of falls in challenged subjects and patients.

## Discussion

In this work, we developed a new low-cost integrated system for presenting postural and visual challenges and measuring static equilibrium including a simple, cheap, widely available balance measurement system – the WBB, a foam pad and an Oculus Rift. The approximate costs in US dollars are Wii board $100; iPod touch $200; Oculus Rift $350 + computer $1000. We have shown how this combination is useful not only for measuring balance but also for possible habituation, and so may be useful in rehabilitation. This easy-to-use system is interesting because:

1. It gave reliable and similar data compared to the Equitest platform in comparable conditions.
2. It provides a test of the difficulty of subjects and patients to maintain balance without vision and with distorted proprioception. Most healthy subjects could stand upright. However, in such conditions, seniors and patients may exhibit postural oscillations but without falling.
3. Is maintaining balance an automatic process? Or does selective attention play a role? The use of visual distractors is the best tool to answer that question.

Here, we quantified balance performance in the face of challenges to balance. Sensory stimuli were similar to the ones used in the SOT on the Equitest: visual, vestibular, and proprioceptive sensory inputs. The SOT is a commonly used test in clinical practice that challenges and evaluates postural control by determining scores for several conditions: eyes open, eyes closed, sway-referenced platform, and sway-referenced vision. These scores are summarized in Table 3.

**Table 3:** **Sensory (visual, proprioceptive, and vestibular) scores calculated by the Equitest**.

Score	Comparison	Functional relevance
Somatosensory (SOM)	Condition 2	Subject’s ability to use input from the somatosensory system to maintain balance
	Condition 1	
Visual (VIS)	Condition 4	Subject’s ability to use input from the visual system to maintain balance
	Condition 1	
Vestibular (VEST)	Condition 5	Subject’s ability to use input from the vestibular system to maintain balance
	Condition 1	
Preference (PREF)	Condition 3 + 6	The degree to which a subject relies on visual information to maintain balance, even when the information is incorrect
	Condition 2 + 5	

*The conditions were: Condition 1: the subject is asked to stand still while maintaining his eyes open. Condition 2: the previous procedure is repeated, but with eyes closed. Condition 3: the cabin moves adaptively following subject’s movements: sway-referenced vision. Condition 4: the base of support adapts at the movements of the subject while having his eyes open. Condition 5: same as before, with eyes closed. Condition 6: the base of support and the cabin move synchronized: vision and proprioception are sway-referenced*.

However, several differences between our system and Equitest should be noted:

– The foam rubber pad modifies the proprioceptive input in a way which is different from the sway-referenced condition of the Equitest.
– The visual inputs used in this pilot study are different from the sway-referenced vision used in the Equitest. In the Equitest, the visual information is false because the visual world is stationary.

Moving visual scenes have been known to induce postural sways in human subjects (11, 12). A huge number of moving 2D visual stimuli have been employed but they are all dependent on the frequency of the scene movement. In our test, subjects receive powerful and unpredictable visual inputs such as moving VR scenes.

On the WBB without foam, the moving visual scene at the maximal amplitude (1.0) induced a fall in only 20% of healthy subjects. On that firm surface, the postural control system has stable sensory inputs from proprioceptive cues to maintain stability. Proprioceptive cues give a body referenced in space relative to the support surface and allow the subject not to be perturbed by visual distractors, or at least to suffer much less perturbation than in the absence of such cues. So, they can easily maintain their equilibrium because the primary sensory source for information about body orientation in space is held to be proprioceptive (13) and the vestibular system appears to play no part in the perception of sway (14).

That is not true on the WBB with foam. In eyes closed condition, the primary source of sensory information shifts from proprioceptive to vestibular because vestibular inputs provide absolute information about the body’s orientation (13). In moving-scene conditions on the foam, proprioceptive/vestibular cues and visual cues give discordant sensory information of movement. The postural control system is unable to solve this sensory conflict. As soon as subjects step on the foam, they detect the lack of a solid base, and accordingly have to increase their concentration and actively work continuously to maintain a standing position. Consequently, we suggest that visual inputs cannot be correctly processed and were not recognized as false information. We showed that VR with an arbitrary amplitude value of 1.0 on WBB + f induced falls in 90% of healthy subjects because of discordant disturbances of the visual and proprioceptive/vestibular systems. This is the reason we chose VR at an amplitude of 0.5 for the initial procedure of habituation.

Time to fall and percentage of falls are both important parameters because they provide different information. Percentage of falls is useful for evaluating the strength of stimulation, as illustrated in the figures. If all subjects fall, the stimulation is too strong to be used on patients suffering from imbalance. Time to fall is useful to evaluate the subject’s sequential performance over time and to follow the patient during rehabilitation or treatment. Moreover, time to fall is probably useful to evaluate the risk of falling: subjects who fall immediately may have higher risk of falling than subjects who resist for a longer time before falling.

Finally, cognitive resources play a key role in maintaining postural stability. It has been shown that older adults recruit cognitive resources to compensate for age-related decline in sensory function (15, 16). Maintaining balance requires processing of three sensory inputs. Based on the dual-task paradigm, we supposed that these sensory inputs need prioritization between themselves. Our system leads to a sensory conflict situation where none of the sensory distractors can be stopped (as opposed to the dual-task paradigm). This system is a single-task (maintaining balance) dual-distractor (foam and moving scene) paradigm. That appears to be the reason we induced falls in healthy subjects. Moreover, the sensory conflict requires prioritization between the sensory cues and so requires mental load. With our visual moving scene, it is possible to increase the amplitude of the stimulus and so to establish an individual threshold at which the sensory conflict cannot be solved by mental processing. It has been shown that mental load is correlated with a fall (17). That is why we suggested that the lower this threshold the higher is the risk of falling.

## Conclusion and Perspectives

We have shown this new system is safe, precise, low cost, and efficient. This new system allows clinicians to obtain objective measures of postural stability and it will help us to better understand the attentional demands required for standing upright. We plan to use it in different cohorts of patients: seniors and vestibular patients at different stages following surgical lesion of the vestibular nerve, to predict the risk of falls. The principle of dual-task performance to predict falls has already been shown as useful in predicting falls when walking (7). However, the published data are heterogeneous, and standardization of test methodology for predicting falls during walking has not been definitively established. Here, the procedure used is easy to standardize with the technology used and described above. More importantly, we hope that new rehabilitation methods will be developed with VR to improve the quality of daily life of patients suffering from dizziness.

## Author Contributions

CW devised the protocol and wrote much of the paper, HM developed the methods, EC tested subjects, wrote much of the paper, conducted the analyses; SR wrote the programs for the visual displays; AB assisted with the writing and production of the paper; IC wrote some of the paper and consulted.

## Conflict of Interest Statement

Hamish G. MacDougall, Ann M. Burgess, and Ian S. Curthoys are currently receiving a project grant (App1046826) from NHMRC of Australia. Hamish G. MacDougall and Ian S. Curthoys are currently receiving grants from the Garnett Passe and Rodney Williams Memorial Foundation; one of these grants helps to pay the salary of Ann M. Burgess. Hamish G. MacDougall and Ian S. Curthoys are unpaid consultants to and have received funding for travel from GN Otometrics. Elodie Chiarovano is in receipt of a doctoral mobility grant from the University of Paris Descartes, and Catherine de Waele and Elodie Chiarovano are also funded by the Grand Audition Society. Stephen J. Rogers has no conflict of interest to declare.

## Supplementary Material

The Supplementary Material for this article can be found online at http://journal.frontiersin.org/article/10.3389/fneur.2015.00164

## Abbreviations

COP, center of pressure; SOT, sensory organization test; VR, virtual reality; WBB, Wii balance board; WBB + f, Wii balance board with foam.
