# Supplementary material for: Maintaining Balance when Looking at a Virtual Reality Three-Dimensional Display of a Field of Moving Dots or at a Virtual Reality Scene
Source: Front Neurol. 2015 Jul 27;6:164. doi: 10.3389/fneur.2015.00164 (PMC4515556; doi:10.3389/fneur.2015.00164)
Supplement: Supplementary file 1 [file Presentation_1.PDF]

Supplementary Material

Maintaining balance when looking at a virtual reality three dimensional display of a field of moving dots or at a virtual reality scene

Elodie Chiarovano[1], Catherine de Waele[1]*, Hamish G. MacDougall[2], Stephen J. Rogers[2], Ann M. Burgess[2], Ian S. Curthoys[2]

[1] Cognition and Action Group – CNRS UMR 8257 – Université Paris Descartes, Centre Universitaire des Saints-Pères, Paris, France
[2] Vestibular Research Laboratory, School of Psychology, University of Sydney, Sydney, NSW, Australia

*** Correspondence:** Dr Catherine de Waele, Cognac G – University Paris Descartes UMR 8257, CNRS, Faculté de Medecine, Paris, France.
catherine.de-waele@parisdescartes.fr

1. Supplementary Data

Attached is a short movie (AVI format) showing an example of the optokinetic stimulus: the three-dimensional display of small moving white spheres on a blue background.
